# Direct estimation of activity concentration in regional voxels with application to ^177^Lu peptide receptor radionuclide therapy

**DOI:** 10.1002/mp.70424

**Published:** 2026-04-05

**Authors:** Johan Gustafsson, Erik Larsson, Katarina Sjögreen Gleisner

**Affiliations:** ^1^ Medical Radiation Physics, Lund, Lund University Lund Sweden; ^2^ Department of Hematology, Oncology, and Radiation Physics Skåne University Hospital Lund Sweden

**Keywords:** lutetium‐177, tomographic reconstruction, quantitative SPECT

## Abstract

**Background:**

Quantitative SPECT in radionuclide‐therapy is limited by partial‐volume effects (PVEs). The implementation of regional voxels (r.v.), estimating mean activity concentrations in regions directly from projections, offers a promising alternative for the geometry‐specification to reduce PVEs.

**Purpose:**

This study aims to demonstrate that activity‐concentration estimation with r.v. is superior to reconstruction with cuboid voxels (cu.v.) with post‐reconstruction partial‐volume correction (PVC) for estimation of activity concentration in ^177^Lu peptide receptor radionuclide therapy (^177^Lu‐PRRT).

**Methods:**

Data originated from one patient administered [^177^Lu]Lu‐DOTA‐TOC with SPECT acquired at 1 d, 4 d and 7 d p.i. stored in list‐mode format (dataset PA), and eight patients given [^177^Lu]Lu‐DOTA‐TATE with SPECT acquired 1 d p.i. (dataset PB). Activity concentration was estimated from reconstruction with cu.v. and using r.v. for both datasets, with multiple noise realizations for PA using bootstrapping. Organ delineation was performed based on CT using the AI tool TotalSegmentator, and tumor delineation made in cu.v. SPECT images. The estimated activity concentration for kidneys, spleen, and tumors from r.v. was compared to that obtained with cu.v. with and without post‐reconstruction PVC. To study the accuracy of activity‐concentration estimates, simulations were performed with the SIMIND Monte Carlo program with patient images used as basis. The sensitivity to misalignments between SPECT and CT was also evaluated.

**Results:**

For both patient and simulated data, activity concentrations estimated with r.v. are higher than those from cu.v., with comparable standard deviations. Mean relative errors for simulated images from PA relative to simulation input at 1 d p.i. reconstructions with r.v. are (−4.6 *± *1.4) %, (3.0 *± *0.5) %, (0.1 *± *0.5) %, and (5.6 *± *1.2) % for tumor, left kidney, right kidney, and spleen, respectively. Corresponding results for cu.v. with post‐reconstruction PVC are (−12.3 *± *2.2) %, (‐4.2 *± *0.6) %, (−7.0 *± *0.5) %, and (‐2.1 *± *1.1) %. For simulated images based on PB, the mean relative errors obtained for r.v. are (−3.1 *± *3.5) %, (1.2 *± *1.2) %, (−1.7 *± *1.1) %, and (2.3 *± *0.8) %, while for cu.v. with PVC they are (−7.9 *± *6.7) %, (‐5.8 *± *1.9) %, (−9.0 *± *1.0) %, and (‐0.7 *± *2.6) %.

**Conclusions:**

Regional voxels are superior to cu.v. for estimation of the activity concentration in organs in ^177^Lu‐PRRT and demonstrates lower sensitivity to misregistration errors. For tumors, r.v. yields lower systematic errors than cu.v. but demonstrates a higher sensitivity to image segmentation errors for volumes below approximately 10 mL.

## 1 INTRODUCTION

Quantitative single photon emission computed tomography (SPECT) has become a central tool for patient‐specific dosimetry in radionuclide therapy. Generally, the task of image‐based quantification is fundamentally different from the task of visualizing radiopharmaceutical distributions, and the optimal settings in how images are processed and analyzed thus differ between them. A major obstacle for accurate estimation of activity concentration from SPECT images is the limited spatial resolution, resulting in the partial‐volume effect (PVE),^1^, ^2^, ^3^ that is, an error in estimated regional mean‐activity concentrations. The correction for PVEs (partial‐volume correction, PVC) is closely related to the segmentation of SPECT images,^4^, ^5^, ^6^ and PVC combined with segmentation tend to be the dominating source‐of‐uncertainty for estimated activity concentrations.^2^ To some extent, PVEs can be mitigated by including spatial‐resolution modeling in the tomographic reconstruction.^7^ The integration of such a model is analogous to an inverse filtering process, aiming to recover frequency components suppressed by limited resolution. However, resolution compensation alone is not sufficient to eliminate the resolution‐induced error because of inevitable loss of information in the projection process. Formally, this is due to the inability to separate solutions that differ by null‐space components,^8^, ^9^ components that can only be recovered if additional information is provided. Typically, such information is the assumption that regions exhibit uniform activity concentration.

The go‐to method for PVC is to apply recovery coefficients (RCs)^10^, ^11^ to the average activity concentration in volumes‐of‐interest (VOIs) in SPECT images. Commonly, such RCs are derived from phantom measurements of spheres as function of volume and, possibly, sphere‐to‐background ratio.^12^ This method has the advantage of being easily accessible. However, it is limited in its ability to account for organ shape,^13^, ^14^ as well as the object‐ and background‐dependent convergence rates associated with the non‐linear nature of iterative reconstruction.^15^, ^16^ In general, more complex PVC methods are required to account for region‐specific shapes,^17^, ^18^ such as the recently proposed methods of Minguez Gabina et al.^14^ and in MIRD Pamphlet 33.^19^ There are also techniques that take the non‐linear properties of reconstruction into account by using perturbation or machine‐learning techniques.^20^, ^21^, ^22^

**FIGURE 1:**
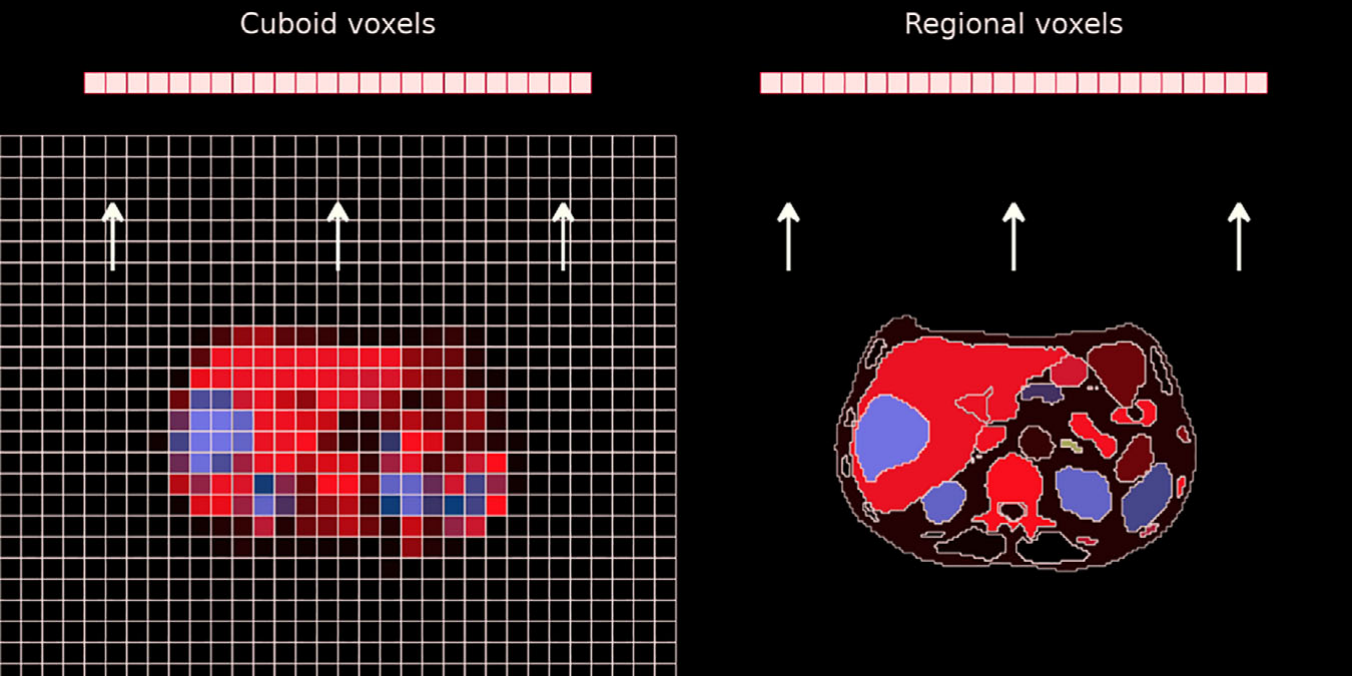
Illustration of reconstruction with cu.v. (left) and r.v. (right). The source distribution described by cu.v. is assumed to be composed of a set of cuboid regions (voxels), while for r.v. the source distribution is described as a set of regions.

An attractive class of PVC methods is to assume region‐wise uniform activity concentration directly in the source estimation, that is, by using regional voxels (r.v.). The r.v. concept is illustrated in Figure 1. These methods address the resolution‐induced information loss by restricting the number of possible solutions to the estimation problem, by using basis functions in the form of VOIs for specifying the source distribution, instead of cuboid voxels (cu.v).^23^, ^24^ A VOI‐based source specification consists of image elements that are tailored to the underlying activity distribution. While the task of reconstruction with cu.v. is to estimate data for the large number of elements defined by the three‐dimensional matrix dimensions, the task when using r.v. is to estimate activity concentrations for (fewer) regions that are aimed at being suited to the problem. While it is technically possible to use r.v. for visualization as an image, this is not the main purpose for this technique. The geometric information shown will always follow that inferred from segmentation step and thus no new information can be obtained in that respect. Thus, r.v., similarly to other PVC techniques, is mainly a tool for quantification and has little role to play in other imaging tasks. Regional voxels have previously been demonstrated useful for estimating regional means provided that the source distribution can be accurately segmented into uniform regions,^25^, ^26^ with an account of the underlying theory detailed by Li et al.^27^ However, the applicability of r.v. has been limited by this requirement for whole‐image segmentation. Some studies have used a combination of r.v. and cu.v. to reduce the need for segmentation,^28^, ^29^, ^30^ or have used a Bayesian framework with fuzzy VOIs to decrease the susceptibility to segmentation errors.^31^ However, since the primary motivation for using r.v. is to provide *á priori* information, an overly generous use of cu.v. will gradually reintroduce PVEs, resulting in degraded estimates. In recent years, the field of automated medical‐image segmentation has advanced considerably through deep‐learning techniques, and full‐body segmentation of CT images is today readily achievable.^32^ This potentially offers a solution for the VOI definition of organs for r.v. Thus, we believe there is reason to explore the potential gain of r.v. in quantitative SPECT.

Evaluation of activity‐concentration estimation using r.v. is challenging, as its performance is closely linked to the accuracy of image segmentation. Hence, it is particularly important to address geometries representative of patients, rather than using stylized phantom geometries or Monte Carlo simulations of simple patient models. An advantage of the r.v. technique is that it provides a direct mapping of mean activity concentrations for all specified regions, thereby eliminating the need for additional PVC. This also offers the possibility to use patient activity‐concentration estimates and CT images as input to Monte Carlo simulations. In this way, we find that quantitative evaluation can be performed solely based on patient data, both concerning the agreement between methods and the quantitative performance.

The aim of this paper is to demonstrate that ^177^Lu activity‐concentration estimation based on r.v. is superior to cu.v. with post‐reconstruction application of RCs for tumor and organ activity‐concentration estimation in ^177^Lu peptide receptor radionuclide therapy (^177^Lu‐PRRT).

## 2 METHODS

### 2.1 Patient data

Patient data were obtained from the START‐NET (EudraCT 2021‐002218‐15) and ILUMINET (EudraCT 2011‐000240‐16) clinical trials for the treatment of neuroendocrine tumors. Data from the START‐NET trial represented one patient with serial SPECT images. Data from the ILUMINET trial came from eight patients, each with a single SPECT image.

The patient from the START‐NET trial was administered 7 375 MBq [177Lu]Lu‐DOTA‐TOC and underwent two‐bed‐position SPECT/CT studies at 26 h, 99 h, and 170 h p.i. A GE Discovery 670 system (GE Healthcare, Haifa, Israel) with 5/8″ crystals equipped with medium‐energy collimators was used with 60 projections over 360° and 45 s acquisition time per projection. Data were stored in list‐mode format. Projections were divided into one‐second bins as 128*×*128 matrices with 4.42*×*4.42 mm^2^ pixels and a 15 % energy window at 208 keV. Thirty‐two bootstrap noise realizations with 45 s per projection were created by random drawing and summation of time bins. This dataset will henceforth be referred to as PA. Low‐dose CT images (tube voltage 120 kV, noise index 45, smart mA in the range 30–120 mA, pitch 1.375, 40 % ASIR, slice thickness 3.75 mm) were acquired for attenuation compensation and anatomical localization.

Patients from the ILUMINET trial were administered nominally 7 400 MBq (range 7 365 MBq to 7 560 MBq) [177Lu]Lu‐DOTA‐TATE and underwent single‐bed‐position SPECT/CT at 1 d p.i. (range 20.0 h p.i. to 23.4 h p.i.). Acquisition parameters followed those for dataset PA but projections were not stored in list‐mode format, and thus did not allow for multiple noise realizations. This dataset will henceforth be referred to as PB.

### 2.2 SPECT‐reconstruction framework

A framework for quantitative SPECT reconstruction was implemented in the IDL language (version 9.0, NV5 Geospatial Solutions, Inc., Broomfield, Colorado, USA). Source code is available at github.com/johan‐ruben. The projector for the iterative reconstruction was implemented as a rotation‐based projector with linear interpolation. For multi‐bed acquisition the projector also accounted for offsets in axial position, that is, the source was projected to each axial position separately and, correspondingly, back‐projection was made for each axial position separately. For attenuation compensation, CT images were rescaled to density using a multi linear‐segment relationship.^33^ Density maps were rescaled to linear attenuation coefficients based on region‐wise assigned material (soft tissue and bone) from a segmentation of the density image using thresholding. Scatter was modeled in the forward‐projector using a variant of the ESSE method.^34^ The difference from the original ESSE method was that the voxel‐wise mean energy of the effective scatter source was computed instead of the mean attenuation coefficient, and the scatter source projected voxel‐wise according to that mean energy. Resolution compensation was modeled as plane‐wise convolution of the source and effective scatter‐source using distance dependent kernels according to the geometric model derived by Metz et al.^35^

Source distributions described by r.v. were implemented as the combination of a three‐dimensional matrix and a region map, where the former described the source distribution in three dimensions and the latter was a three‐dimensional matrix of labelled regions. The purpose of the region map was to describe the geometry of the r.v. Regions not labelled by the region map could optionally be partitioned into cuboid regions with pre‐specified size.

Forward‐ and back‐projection for source distributions described by r.v. were implemented using the same methods as for cu.v., with the addition that values were averaged within regions as part of the back‐projection. To allow for a detailed r.v. description, the source distribution and region map were defined with a finer voxel size than what is typically applicable for SPECT. However, from the perspective of the projector, such a fine voxel size would not contribute to more accurate projection estimates and only lead to deteriorated computational efficiency. For this reason, the source distribution was represented with two voxel‐ and matrix sizes in parallel: one internal representation that conformed to the region map and one external representation with a coarser discretization. From the perspective of the projector, the source behaved as an image in the external representation and thus the corresponding voxel size was termed the emulated voxel size. Practically, the source‐distribution matrix was down‐sampled from the internal representation to the emulated voxel size when retrieved by the projector, and internally up‐sampled from the emulated to the finer voxel size when the back‐projector returned the back‐projected image. Since it was important to preserve the total source activity during the up‐ and down‐sampling processes, the emulated voxel size was restricted to an integer factor of the internal representation. Thus, the down‐sampling was implemented as an averaging over an integer number of voxels, while the up‐sampling consisted of a partitioning of larger voxels into smaller ones. The emulated voxel size was also used for the regions not explicitly labelled by the region map described above.

This implementation strategy allowed for the same forward‐ and back‐projection routines to be used for cu.v. and r.v., the mixing of r.v. and cu.v. within the same source description, and the r.v. to be described with a finer discretization than the emulated voxel size.

### 2.3 SPECT reconstruction with cu.v

Tomographic images described by cu.v. were reconstructed using OS‐EM with five to 40 iterations and ten subsets. For single‐bed‐position acquisitions the matrix size was 128*×*128*×*128 with 4.42*×*4.42*×*4.42 mm^3^ voxels. For dual‐bed‐position acquisitions the matrix size was 128*×*128*×*208 with the same voxel size. No post‐reconstruction filter was applied. Calibration to activity concentration was performed based on SPECT measurement of a cylindrical phantom (volume 5.6 L) filled with a uniform solution of ^177^Lu and reconstructed with the same settings as patient data. A large cylindrical VOI (volume 770 mL) was positioned centrally in the phantom and the reconstructed signal in the VOI related to the known activity concentration in the phantom. Reconstructed images were re‐sampled to 1.105*×*1.105*×*4.42 mm^3^ by subdivision of voxels, as described above. Delineation of organs was based on CT using TotalSegmentator^32^ (version 2.4.0). Tumors were identified manually and delineated using the morphological Chan‐Veese method.^36^, ^37^ For organs in dataset PA, a single VOI template was used, while for tumors the VOIs were delineated for each reconstructed bootstrap realization.

Post‐reconstruction PVC was applied using RCs. These were derived from measurements of spheres described in Roth et al.,^38^ and recovery parameterized as function of volume‐to‐surface ratio η and number of iterations n following

(1) $$\mathrm{RC}\;\left(\eta ,\;n\right)=\frac{1}{1+{\left(\frac{\alpha_{n}}{\eta }\right)}^{\beta_{n}}}\;,$$

where $\alpha_{n}$ and $\beta_{n}$ are parameters determined by non‐linear least‐squares fitting.^39^

### 2.4 Projection‐based activity‐concentration estimation with r.v

Region maps were defined with voxel size 1.105*×*1.105*×*4.42 mm^3^ based on the same set of VOIs as used for image analysis of cu.v. images, and the emulated voxel size of the source set to 4.42*×*4.42*×*4.42 mm^3^. A regional voxel in the form of a background region was defined as voxels not belonging to any other region. The exception was the 25 first and 25 last transversal slices that were left unlabeled. This was to avoid interference from zero‐values in the periphery of the projections caused by the gamma‐camera detector being smaller than the projection matrix. For PA, estimation was performed using OS‐EM with ten subsets and one to eight iterations. Both fixed tumor VOIs over bootstrap realizations and using VOIs delineated for each realization were applied.

### 2.5 Monte Carlo simulated images

Monte Carlo simulations were designed to replicate the patient measurements both in terms of geometry and activity concentrations, enabling evaluation of estimated activity concentrations from images towards those defined by simulation input. Projections for a single bed position were simulated using the SIMIND Monte Carlo program.^40^ Patient activity concentrations obtained from r.v. for datasets PA and PB were used as source maps and the corresponding CTs rescaled to density used as phantoms. These simulated images based on data from PA and PB will be referred to as SA and SB, respectively. Arms and couch were excluded from the phantoms and acquisitions simulated with detector orbits from the corresponding patient studies. A large number of histories were used to achieve essentially noise free projections, projections were scaled to the sought activity and time per projection, and Poisson‐distributed noise added. For dataset SA, 32 noise realizations per time‐point were created, while a single noise‐realization per patient was created for dataset SB. Activity‐concentration estimation with cu.v. and r.v. were performed as for patients. VOIs for organs were derived from simulation input. Two VOI strategies were applied for tumors, either using the masks from simulation setup or using VOIs obtained from semi‐automatic delineation in cu.v reconstructions, as for patient images. Images with r.v. geometry were reconstructed for both types of VOIs.

For post‐reconstruction PVC, a NEMA PET body phantom with six spheres (volume range 1.2 mL to 113.1 mL) was simulated with a detector orbit retrieved from previous phantom measurements.^41^ Tomographic images with cu.v. were reconstructed and a recovery curve fitted according to Equation 1.

### 2.6 Evaluation

#### 2.6.1 Patient images

The mean activity concentration and standard deviation over bootstrap realizations were computed for tumors, kidneys, and spleen for r.v. and cu.v. estimates from dataset PA. Estimates for cu.v. were studied with and without post‐reconstruction PVC using RCs. The RCs were applied according to^11^

(2) $$C_{\mathrm{cu}.v.,\mathrm{pvc}}=\frac{C_{\mathrm{img}}-C_{\mathrm{bkg}}\left(1-\mathrm{RC}\left(\eta ,n\right)\right)}{\mathrm{RC}\left(\eta ,n\right)}\;,$$

where $C_{\mathrm{img}}$ is the activity concentration estimated directly from the SPECT image and $C_{\mathrm{bkg}}$ is the background activity concentration. The latter was estimated from a background VOI obtained as the logical difference between successive dilations of the object VOI. The surface‐to‐volume ratio of the object η was estimated from the VOI volume and the area of a polygon surface rendered from the VOI mask using the Shade_Volume routine in the IDL distribution.

For dataset PB, the relative difference between estimates from r.v. and cu.v. was calculated as

(3) $$\delta =2\frac{C_{r.v.}-C_{\mathrm{cu}.v.,\mathrm{pvc}}}{C_{r.v.}+C_{\mathrm{cu}.v.,\mathrm{pvc}}},$$

where $C_{r.v.}$ is the activity concentration estimated from r.v. and $C_{\mathrm{cu}.v.,\mathrm{PVC}}$ is the activity concentration estimated from cu.v. with post‐reconstruction PVC. Tumors located in the periphery in the field‐of‐view were excluded from analysis.

#### 2.6.2 Monte Carlo simulated images

The relative error in estimated activity concentration for noise realization i in dataset SA was calculated according to

(4) $$\varepsilon_{i}=\frac{C_{\mathrm{est},\;i}}{C_{\mathrm{ref}}}\;-1,$$

where $C_{\mathrm{est},i}$ is the estimated activity concentration and $C_{\mathrm{ref}}$ is the concentration defined in the simulation. The relative error was determined for estimates from r.v. and cu.v., where the latter was both with and without post‐reconstruction PVC using Equation 2. The mean relative error and standard deviation were calculated over noise realizations for tumors, kidneys, and spleen.

For dataset SB, the relative activity‐concentration error was evaluated as a function of volume for the same structures as for SA. Tumors located in the periphery of the field‐of‐view were excluded from analysis.

#### 2.6.3 Sensitivity to misalignment

Since the r.v. technique relies on structural information inferred from prior segmentation of CT, and the techniques with cu.v. similarly rely on the application of VOIs derived from CT for extracting averages post reconstruction, it was considered important to assess its sensitivity to misalignment between SPECT and CT. The sensitivity of activity‐concentration estimates for normal organs (kidneys and spleen) to misalignment between SPECT and CT for the three different estimation methods was performed using dataset SB. The CT was translated by −1.0 cm to +1.0 cm in the x‐ y‐ and z‐directions in steps of 0.2 cm before segmentation with TotalSegmentator and the density‐map was translated by the same amount in the tomographic reconstruction. The relative error (Equation 4) in estimated activity concentration was studied as function of offset.

## 3 RESULTS

### 3.1 Patient images

**FIGURE 2:**
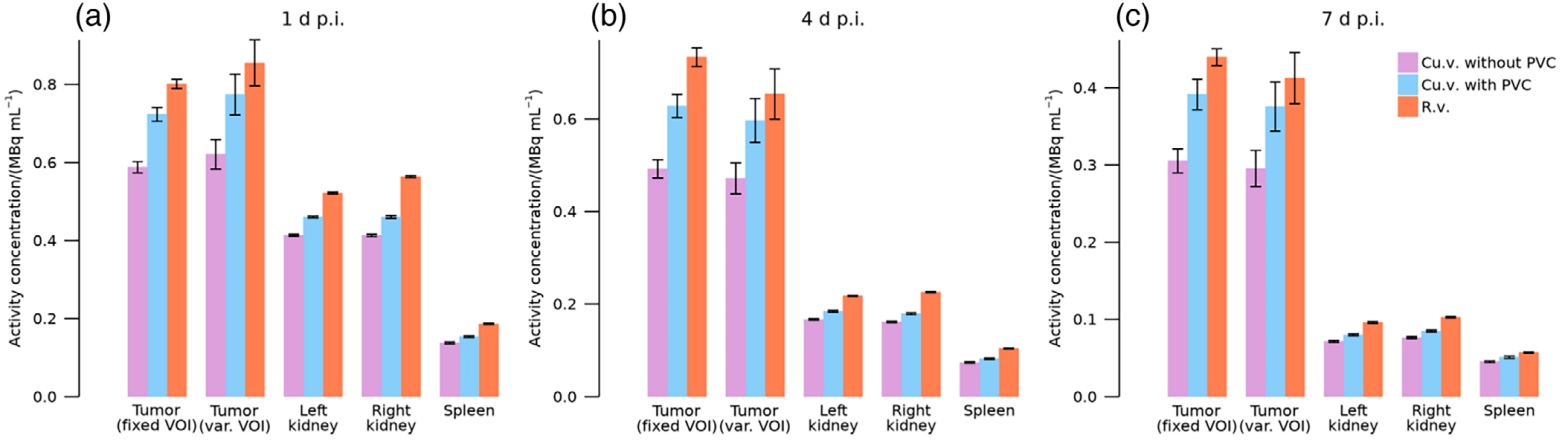
Mean estimated activity concentration and standard deviation over bootstrap realizations for r.v., cu.v. with PVC, and cu.v without PVC. Concentrations are shown for tumor, kidneys, and spleen in patient dataset PA, at imaging time‐points (a) 1 d, (b) 4 d, (c) 7 d. Results for tumor are reported both when the VOI is kept fixed over bootstrap realizations (fixed VOI) and when the VOI is specific for each bootstrap realization (var. VOI).

Recovery as function of volume‐to‐surface ratio is shown in Figure S1. Only one tumor is identifiable for the patient in dataset PA. Estimated activity concentration as function of number of iterations are presented in Figures S2 and S3, showing that estimates for r.v. stabilize at a lower number of iterations than for cu.v. Stable estimates are obtained at eight iterations (maximum relative change between seven iterations and eight iterations of 0.2 %) and 40 iterations (maximum change between 35 iterations and 40 iterations of 0.8 %) for r.v. and cu.v. respectively. Further results are thus presented for these numbers of iterations only. Figure 2 shows mean activity concentrations and standard deviations for data set PA. Systematic differences are observed between the three methods (e.g., the tumor for fixed VOI 1 d p.i.: (0.802 ± 0.012) MBq mL^−1^, (0.713 ± 0.018) MBq mL^−1^, and (0.588 ± 0.014) MBq mL^−1^ for estimates from r.v., cu.v. with PVC, and cu.v. without PVC, respectively), with the highest estimates obtained when using r.v. Standard deviations over bootstrap realizations are similar across methods and negligible for organs. For tumor, the dispersion is small when using fixed VOIs compared with systematic differences between methods, and becomes larger when using VOIs specific to each bootstrap realization. This indicates the sensitivity to segmentation errors associated with image noise.

**FIGURE 3:**
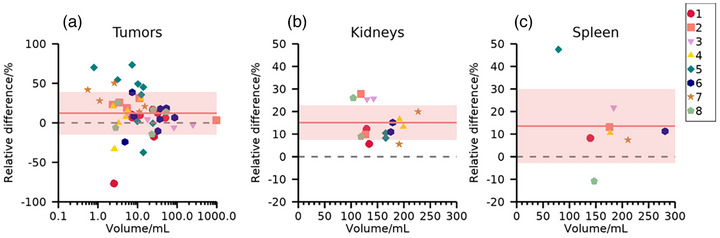
Relative differences (Equation 3) between estimated activity concentrations using r.v. and cu.v. with PVC for (a) tumors, (b) kidneys, and (c) spleen for the eight patients in dataset PB, indicated by colored markers. Horizontal lines indicate zero deviation (dashed) and the mean difference across data points (solid) with one standard deviation indicated by shaded bands.

Figure 3 shows the relative differences in estimated activity concentrations between r.v. and cu.v. for dataset PB. As for PA, r.v. yields on average higher activity concentrations than cu.v. with PVC, indicated by the positive deviations. Kidneys demonstrate lower dispersion of relative differences compared with tumors and spleen. The dispersion for tumors increases as the volume decreases.

### 3.2 Monte Carlo simulated images

**FIGURE 4:**
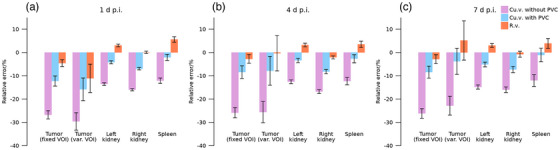
Mean relative errors (Equation 4) in estimated activity concentration for dataset SA at the three imaging time‐points p.i., (a) 1 d, (b) 4 d, and (c) 7 d. Results are shown for estimates from r.v. and cu.v. with or without post‐reconstruction PVC, and represent the mean and standard deviation over noise realizations for tumors, kidneys, and spleen. Results for tumor are shown for both VOIs kept fixed from simulation input (fixed VOI) and for VOIs defined in each noise realization of cu.v. images (var. VOI).

Figure 4 shows mean relative errors of estimated activity concentrations from dataset SA, using r.v. and cu.v. with or without post‐reconstruction PVC. As for patient data (Figure 2), estimates for r.v. are higher than when using cu.v. with similar standard deviations over noise realizations. The mean relative errors obtained with r.v. are within 10 % across all tissues and generally yield similar or better accuracy than corresponding cu.v. reconstructions. The dispersion and differences between time‐points are larger when using tumor VOIs defined separately for each noise realization, compared with using a fixed VOI, as also seen for PA. Figures S4 and S5 show relative errors as a function of number of iterations.

**FIGURE 5:**
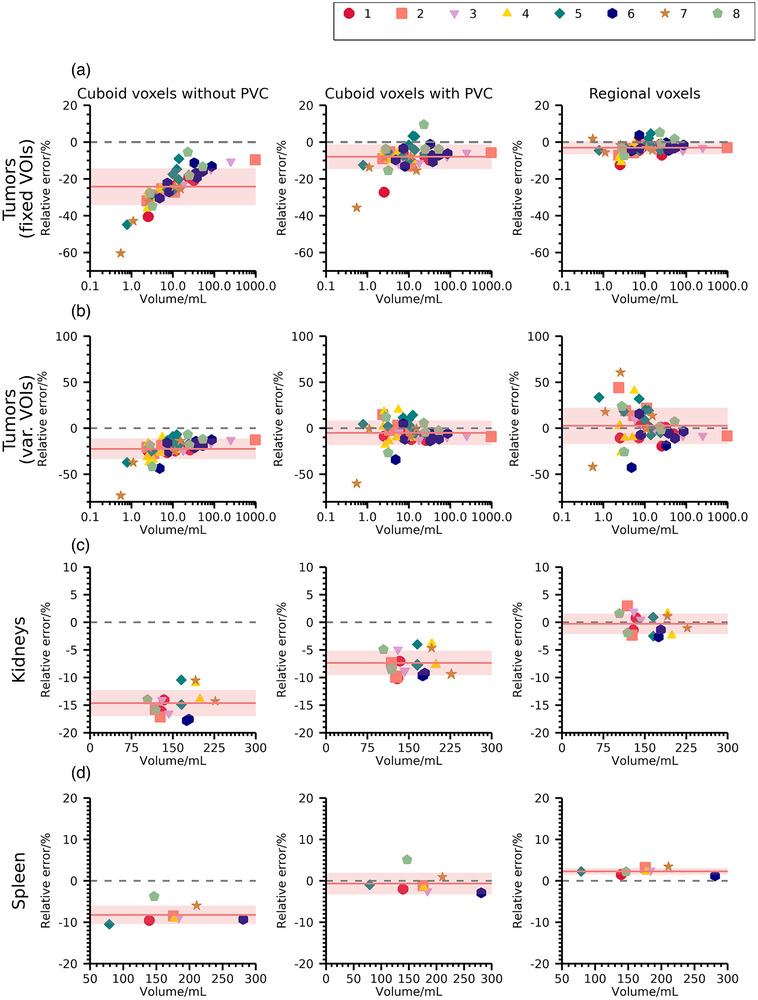
Relative errors of estimated activity concentrations (Equation 4) for dataset SB using r.v. and cu.v. with or without post‐reconstruction PVC, for (a) and (b) tumors, (c) kidneys, and (d) spleen. Results for tumors are reported both when VOIs are kept fixed from simulation input (fixed VOIs, a) and when VOIs are delineated in each noise realization of cu.v. images (var. VOI, b). Horizontal lines indicate zero deviation (dashed) and mean errors across data points (solid) with one standard deviation indicated by shaded bands.

Relative errors in estimated activity concentrations for dataset SB are shown in Figure 5. Estimates from r.v. yield mean errors across data points that are close to zero for all investigated structures and generally demonstrate better accuracy and precision than cu.v. reconstruction, also when including post‐reconstruction PVC ((−3.1 ± 3.5) % versus (‐7.9 ± 6.7) %, (1.2 ± 1.2)  % versus (−5.6 ± 1.9) %, (1.7 ± 1.1) % (−9.0 ± 1.0) %, and (−2.2 ± 0.8) % versus (0.7 ± 2.6) % for tumors, left kidney, right kidney, and spleen, respectively). When using VOIs derived from SPECT images for tumors, r.v. yields increasing errors for volumes below approximately 10 mL, which are not as pronounced for cu.v. estimates (global mean and standard deviation: (3 ± 20) % versus (‐5 ± 13) % for r.v. and cu.v.).

### 3.3 Sensitivity to misalignment

**FIGURE 6:**
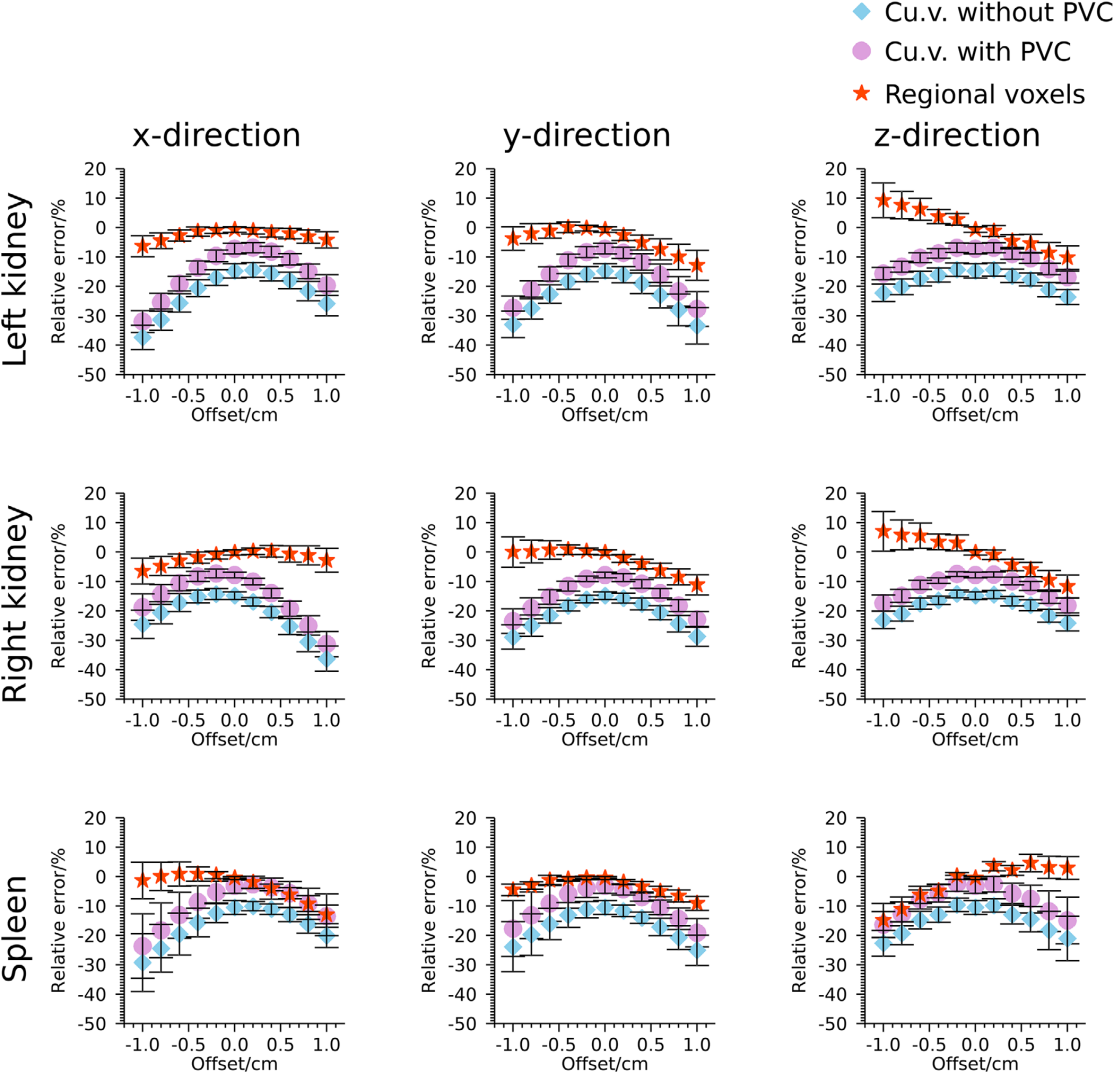
Relative error in activity concentration (Equation 4) as function of offset between CT and SPECT in x‐, y‐, and z‐directions for dataset SB. Symbols indicate means and whiskers indicate ± one standard deviation over the eight phantoms.

Figure 6 presents relative errors in estimated activity concentration as function of offset between SPECT and CT for the eight phantoms in dataset SB. For cu.v. the misalignment produces increasingly pronounced underestimates when the offset increases from zero. Regional voxels are in most cases equally or less sensitive.

## 4 DISCUSSION

Quantitative SPECT is an increasingly utilized tool for patient‐specific dosimetry in radionuclide therapy. PVEs are known to be a major limiting factor for quantitative accuracy, and the *de facto* standard method for its correction has become the application of RCs post‐reconstruction. However, the non‐linearity of modern SPECT‐reconstruction methods^15^, ^16^ leads to geometry‐dependent recovery, which limits accurate post‐reconstruction PVC. On the other hand, resolution modeling within the tomographic reconstruction is a linear process that can be derived from basic geometric collimator properties.^35^ We find that quantification based on the combination of resolution modelling in projection space and the use of r.v. to describe the source distribution is a better strategy than the standard method. A key observation to this approach is that single‐voxel estimates in SPECT are not quantitatively meaningful.^42^, ^43^, ^44^ In practice, all deduced data have to be averages over larger regions, and the need of delineating regions is already inherent to the activity‐concentration estimation‐problem. Thus, the use of r.v. does not introduce additional assumptions than is already the case with cu.v. reconstruction combined with PVC.

The de‐facto standard method for PVC in ^177^Lu‐quantitative SPECT is the application of RCs derived from prior phantom measurements.^45^, ^46^, ^47^, ^48^ While this method provides a first‐order correction of the estimated activity concentration, it is not yet clear how this method should best be implemented in relation to the patient geometry and the settings used for SPECT reconstruction. One aspect raised has been the need of considering the object‐to‐background ratio in the correction.^11^ For the current study, correction for spill‐in from background was included under the assumption of a linear translation‐invariant system, although the high tumor‐to‐background ratio for PRRT^12^, ^49^ makes this spill‐in correction small. Another aspect is the recovery dependency on object shape, as considered in by for example, Marquis et al.^11^ In the present paper, this was managed by using the volume‐to‐surface ratio, a parametrization that has been demonstrated to be more relevant than volume alone.^13^, ^14^ Notably, much of the theoretical issues have been discussed under the underlying assumption of a linear translation invariant system. In practice, the applicability of RCs is inherently limited by the non‐linear nature of modern iterative reconstruction methods in combination with the distance‐dependent spatial resolution of gamma cameras.^15^ Inclusion of resolution compensation in the reconstruction tends to enhance the problems associated with non‐linearity, while omission of such compensation instead results in a spatial resolution that varies over the reconstructed field‐of‐view.^15^ To some extent, the effect of non‐linearity can be counteracted by using a large number of image updates,^49^ such as the 400 updates (40 iterations times 10 subsets) employed herein.

Results for patient data in Figures 2 and 3 demonstrate moderate systematic differences between r.v. and cu.v. for organs, and moderate to large systematic differences for small tumors. Meanwhile, the noise properties of r.v. and cu.v. methods are comparable. Simulations in Figures 4 and 5 for organs demonstrate similar systematic trends, where results from r.v. show lower mean errors than those obtained for cu.v. Thus, the systematic differences observed for patients (Figures 2 and 3) result from underestimations for cu.v., despite the use of post‐reconstruction PVC. For tumors, simulated data (Figure 5) show that r.v. performs excellently when VOIs agree with the regions defined from simulation input, while cu.v. with PVC yields increasingly pronounced underestimates for volumes below approximately 10 mL. When instead delineating tumor VOIs based on each noise realization, considerable random errors result from r.v. for volumes below approximately 10 mL, while cu.v. with PVC is more stable. Thus, the way segmentation errors propagate into errors in activity concentration differs between r.v. and cu.v., where cu.v. with PVC demonstrates a bias for small volumes, while r.v. exhibits larger sensitivity to segmentation errors for small volumes. Together, these results highlight the importance of considering the combination of errors introduced by image segmentation and PVE for quantitative accuracy. With respect to tumors, the prioritization between bias and precision in the quantification of individual tumors may depend on the application. For cases where individual tumors are being examined avoiding imprecision may be preferable, while for examination of averages across a multitude of tumors, the avoidance of bias is more important.

An underlying assumption of the r.v. approach is that the activity concentration is uniform in each defined region. Meanwhile, it is well‐known that the uptake in RNT is non‐uniform for both tumors^50^ and organs,^51^ and thus the assumption of uniformity will never be an exact reflection of reality. However, the practical relevance of non‐uniformity is a question of spatial scale, and with respect to SPECT imaging, non‐uniformity has to be macroscopic to pose an issue. The effect of non‐uniformity on estimates with r.v. for tumors in ^223^Ra therapy for castration‐resistant prostate cancer was studied by Li et al.^27^and found to be modest. The qualitative agreement between patient studies and simulations in the current study indicates a small to moderate effect for the tumors and organs in ^177^Lu‐PRRT, although this is a problem where further studies are warranted.

A limitation to this study is that the effects of segmentation errors for organs have not been studied. For patient data, organ segmentation was based on CT images where multiple noise realizations were not available. Thus, the only source of variability of the estimated activity concentration is the effect of quantum noise in SPECT projections and its propagation to the reconstructed images. Likewise, for simulations, the VOIs applied for organs essentially corresponded to the regions defined in the simulations, making the study somewhat idealized in this respect. However, the sensitivity to misalignment between SPECT and CT was addressed and r.v. was found to be equally or less sensitive than the methods based on cu.v. Somewhat counterintuitively, r.v. resulted in both under‐ and overestimation of the activity concentration when offsets were introduced, while a non‐zero offset as expected yielded underestimation for methods based on cu.v. The r.v. approach, using direct estimation of activity concentration in regions, means that the distribution of activity concentration automatically follows the defined VOIs, and thus there will not be an automatic loss of activity due to VOI mismatch. With respect to tumor delineation, we opted for a published and available semi‐automatic method, which had the advantage of exposing the reconstructed images to a realistic range of segmentation errors. However, we cannot claim the segmentation strategy to be optimal and find that further development of tumor delineation in SPECT is warranted.

## 5 CONCLUSIONS

Activity‐concentration estimation using r.v. combined with whole‐body segmented CT images provides a solid basis for quantifying ^177^Lu activity concentration. Regional voxels offer better accuracy and precision for organs compared to cu.v. For tumors, the results for r.v. are promising but strongly associated with the accuracy of image segmentation for small volumes.

## CONFLICT OF INTEREST STATEMENT

The authors declare no conflicts of interest.

## Supporting information



Supporting information
