# Supplementary material for: Direct estimation of activity concentration in regional voxels with application to 177Lu peptide receptor radionuclide therapy
Source: Med Phys. 2026 Apr 5;53(4):e70424. doi: 10.1002/mp.70424 (PMC13051038; doi:10.1002/mp.70424)
Supplement: Supplementary file 1 — Supporting information [file MP-53-0-s001.pdf]

Supplement to: Direct estimation of activity concentration in regional voxels with application to ^{177}Lu peptide receptor radionuclide therapy

Supplemental figures

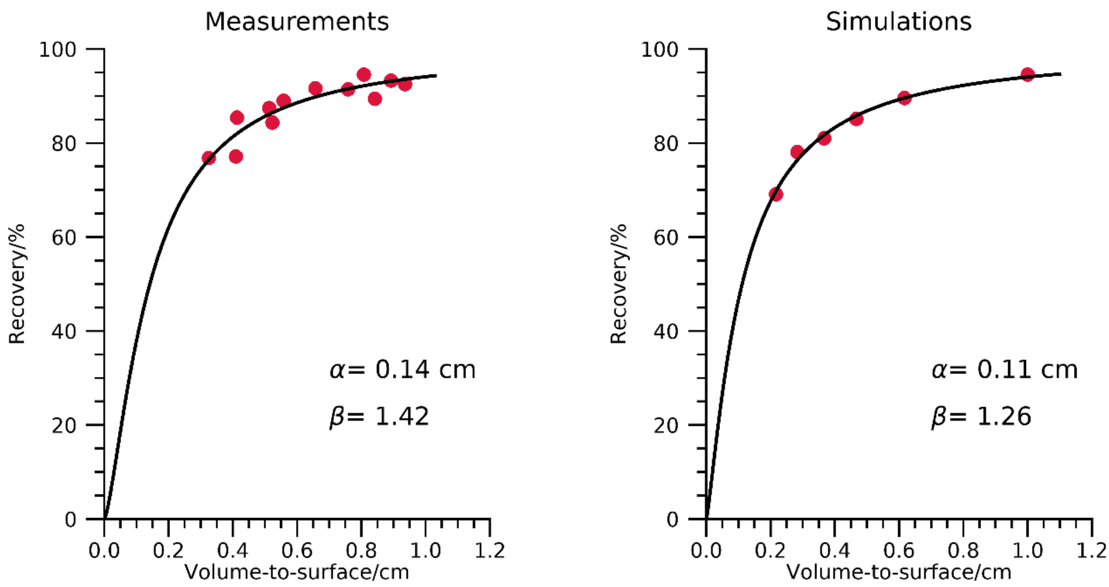

Figure S1: Recovery as function of volume-to-surface ratio at 40 iterations for measurements and simulations.

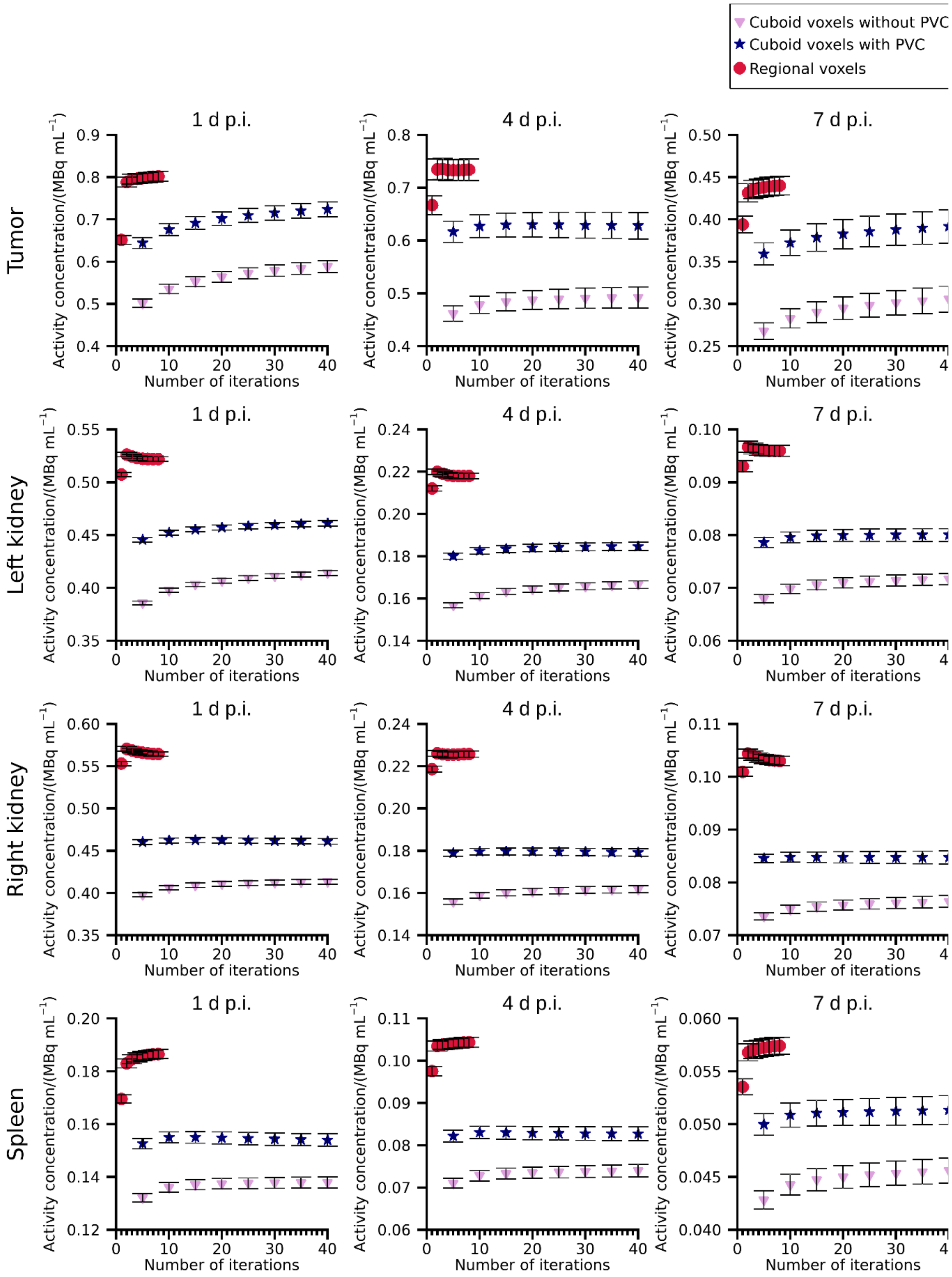

Figure S2: Estimated activity concentration in tumors, kidneys and spleen for cubic and regional voxels as function of number of iterations. Symbols indicate means and bars indicate one standard deviation over bootstrap realizations. The VOIs are kept fixed over bootstrap realizations.

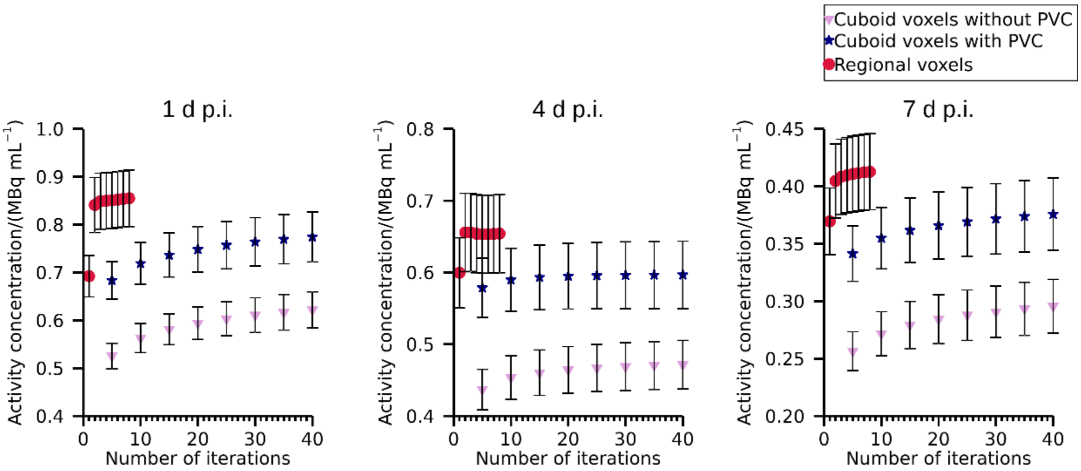

Figure S3: Estimated tumor activity concentration for cubic and regional voxels as function of number of iterations. The VOIs are specific to each bootstrap realization.

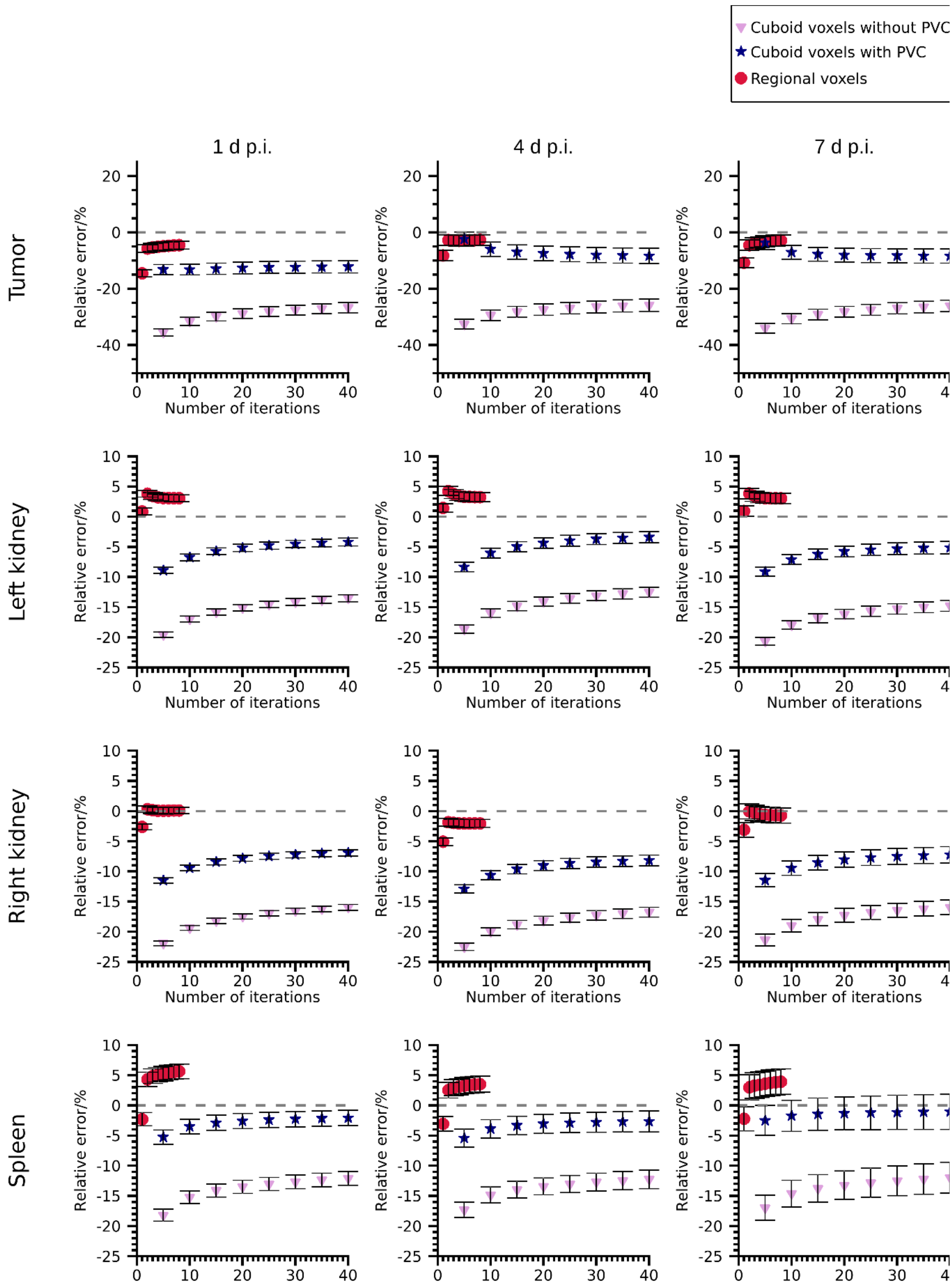

Figure S4: Relative errors in estimated activity concentrations for simulated images. Symbols indicate mean relative errors and bars indicate one standard deviation. VOIs are derived from simulation input.

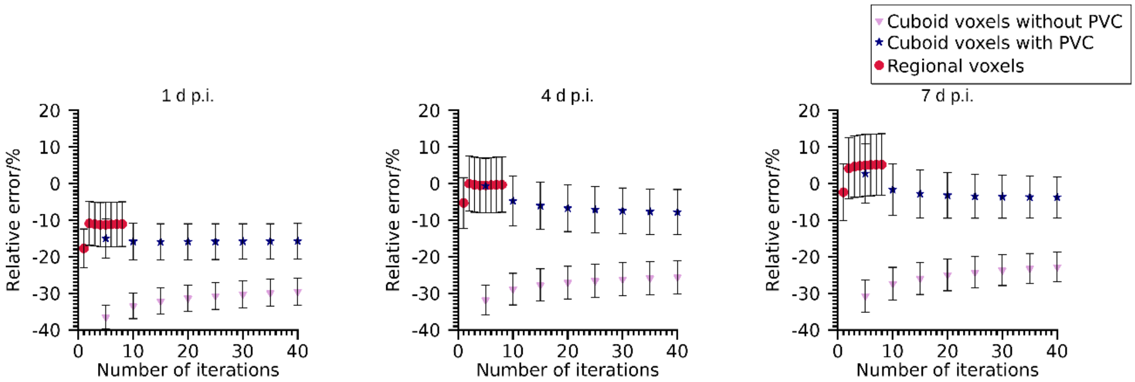

Figure S5: Relative errors in estimated tumor activity concentrations for simulated images from data-set A when using VOIs derived from SPECT images for each noise realization. Symbols indicate mean relative errors and bars indicate one standard deviation.
